# Anthropometric study using three-dimensional pelvic CT scan in sex determination among adult Indonesian population

**DOI:** 10.1007/s12024-022-00526-w

**Published:** 2022-09-14

**Authors:** Rosy Setiawati, Paulus Rahardjo, Ika Ruriana, Giuseppe Guglielmi

**Affiliations:** 1 Radiology Department, Faculty of Medicine, Universitas Airlangga, Surabaya, Indonesia; 2 Department of Clinical and Experimental Medicine, School of Medicine, Foggia University, Foggia, Italy; 3 Department of Radiology, School of Medicine, Foggia University, Foggia, Italy

**Keywords:** Sex determination, Sexual dimorphism, Anthroprometric study, Pelvic 3D CT scan

## Abstract

This study aims to determine pelvic anthropometry characteristics and logistic regression formula for adult sex identification obtained from adult three-dimensional pelvic computed tomography images. This study was an observational analytical study with retrospective regression and cross-sectional approach. The population was all patients at Radiology Installation of Dr. Soetomo General Academic Hospital as referral hospital in East Indonesian region, from September to December 2019 who underwent 3D pelvic CT examination. Then, age distribution and pelvic measurements data were obtained. In this case, statistical analysis was conducted for all the data obtained. A number of 204 samples were included in this study. All radiologic components were also significantly different between sexes (*p* < 0.05) except for transverse diameter of sacral segment (*p* = 0.180). Moreover, the conjugate pelvic inlet diameter (CPID), the left innominate height (LIH), and sub pubic angle (SPA) showed significant values for regression formula to determine an adult’s sex using 3D pelvic CT. The calculation result > 0 is a prediction for female while < 0 is a prediction for male. From logistic regression model calculation, a high validity value (91.05%) was found with 100% sensitivity to identify male sex and 81.1% specificity to identify female sex. There were differences on radiometric variable characteristics in pelvic anthropometric study among adult Indonesians at Dr. Soetomo General Academic Hospital, Surabaya. The estimated values of pelvic measurements using 3D CT images could develop a pelvic model with a regression formula with high accuracy value using CPID, LIH, and SPA values.

## Introduction

Sex identification on remaining human bone is the first step to do in helping forensic anthropologists to identify a person [1]. The accuracy of sex identification depends on bone components that are analyzed and the technique used. Forensic anthropology is a branch of applied physical anthropology that combines a variety of modified scientific techniques and skills from many scientific disciplines and is used to answer medico-legal related questions. Some of these techniques are quantitative and can be tested empirically, e.g., sex and age estimation technique from unidentified bone remains [2, 3].

In adults, the coxa is the most reliable indicator of sex because of its sexual dimorphism. Each population must have a specific identification standard [4]. Os coxae shows a broadly common pattern of sexual dimorphism across many regions in the world, and this pattern of pelvic sexual dimorphism appeared in early modern humans, approximately 100–150 years ago [5].

Accurate estimation of sex for adults is affected by the state of the individual preservation of bones, their degree of expression of sexual dimorphism, and methods employed. In some instances, metric methods are better than morphological ones [6]. For these reasons, a study which demonstrates the importance of an objective method by utilizing radiology technology in sex determination is needed. Although visual or morphological techniques are a quick way of assessment, they have the disadvantage of being highly subjective, requiring experienced observers and often not being able to guarantee accuracy. Without quantifiable data, there are clear implications for determining conclusive results that even the method cannot be used as a basis of reference [7].

Conventionally, anthropologists have relied on metric and non-metric observational analyses of actual bones [3]. One of the oldest methods known in anthropology is Procrustes analysis. Generalized Procrustes analysis standardizes the landmark configurations for variation in position, scale, and orientation. The resulting Procrustes shape coordinates represent the shape of the pelvis only, whereas information about the overall pelvis size is represented by the centroid size of the landmark configurations. They performed a principal component analysis (PCA) of the Procrustes shape coordinates, jointly for males and females. The dependence of pelvis shape on body size was estimated by multivariate linear regression of the Procrustes coordinates on body height, separately for males and females. However, these matrices must first be back-converted into an object-by-variable table by principal cordinates analysis (PCoA), non-metric multidimensional scaling (NMDS), or another suitable ordination method. In general, its use seems desirable since the analysis of shape differences and landmark configuration will increase the likelihood of detecting more subtle local patterns, but the main disadvantage in this analysis is the increased computer time necessary to compute the necessary parameters [8, 9].

Over the past decade, modern cross-sectional imaging techniques have revolutionized forensic medicine. Virtual anthropology obtained by the 3D imaging techniques such as computer tomography (CT) allows us to visualize almost every anatomical and pathological bone structure with high resolution and quality. Multi-slice computed tomography (MSCT) is becoming more and more widely used for post-mortem examinations. CT provides data sources to examine modern human variation quantitatively when expanding the resources for osteological assessment to researchers. These studies have shown significant improvements in accuracy and reproducibility over conventional linear methods of constructing a person’s biological profile [10, 11].

Various attempts have been made to be able to “metricize” or measure non-metric properties accurately in several body areas [11, 12]. More objective data for sex estimation might allow very accurate results along with metrication of certain non-metric pelvis areas [1]. Moreover, other pelvic indices such as those used in clinical medicine might be used to supplement and give additional measurements in anthropological assessment. In adults, pelvic bones are the most reliable indicator of sex because of its sexual dimorphism. Each population must have a special identification standard [3]. This study aims to determine pelvic anthropometry characteristics obtained from 3D pelvic CT in identifying sex among adult Indonesians in Dr. Soetomo General Academic Hospital, Surabaya, Indonesia.

## Materials and methods

### Study design

This study was an observational analytical study with retrospective regression and cross-sectional approach. This study was approved by Medical Research Ethic Committee of Dr. Soetomo General Academic Hospital, Surabaya, for ethical clearance with registration number 0106/LOF/301.4.2/VIII/2020. All participants included had given their written informed consent to participate in this study. Moreover, the population was all patients in Radiology Installation from September to December 2019 who underwent 3D pelvic CT examination. All methods were carried out in accordance with relevant guidelines and regulations.

### Sample of study

The sample of this study was 3D pelvic CT data which obtained from consecutive sampling that suited to the inclusion and exclusion criteria. The inclusion criteria were MIP and VR image reconstruction of 3D pelvic CT images of patients in the Radiology Installation, both male and female patient’s age were more than 18 years old, and non-pregnant women. On the other hand, the exclusion criteria in this study was pelvic CT images of patient’s pelvis and sacrum which had some pathological conditions, e.g., bone implant, chronic disease, bone fracture, osteoporosis, that might interfere the measurement.

### Study procedure

Pelvic CT scan examinations in the form of digital data served as study material. Furthermore, radiological examination tool used a 16-slice Siemens SOMATOM CT scan machine in Radiology Installation of Dr. Soetomo Surabaya General Academic Hospital. The parameters that have been used in axial pelvic CT scans in this study were 120 kV, 140 effective mAs, 1.5 mm of slice collimation, 5.0 mm of slice width, 24.0 mm feed/rotation, 0.5 s rotation time, as well as the effective dose for male: 3.3 mSv and female: 4.8 mSv. 3D CT image began with the acquisition and reconstruction of axial image data. Furthermore, axial images were sent to the workstation (Siemens, Leonardo, Erlanger, Germany). MIP and VR images were obtained using reconstruction procedure on axial image. We obtained 3D images in optimal resolution and spatial resolution in all of the patients. The pelvic CT axial images were reconstructed to produce 3D pelvic CT images that were presented as DICOM (Digital Imaging and Communications in Medicine) images. Each patient’s pelvic data was imported into Mimics software (Materialise, Leuven, Belgium) in DICOM format; the “CT Bone Segmentation” tool is used [13, 14].

All demographic data were obtained from medical records. The age distribution and pelvic measurements data were obtained as well in Table 1 [3]. Blinded pelvic measurements were performed by two observers, musculoskeletal radiologist consultants with more than 10 years of experience. Multiple pelvic measurements of each variable by observer were then obtained and determined the mean of radiometric values. In the inter-observer error test, the results of 10 measured specimens from each radiologist were compared. The inter-observer agreement was evaluated by the calculated Cohen’s kappa coefficient.

**Table 1:** Pelvic measurement definition included in this study

**No.**	**Measurement**	**Definition**
1.	Anterior breadth of the sacrum (ABS)	The maximum transverse point of the sacrum on the anterior orientation of the auricular surface
2.	Anterior height of sacrum (AHS)	Distance between sacrum/coccygeal margin and sacral promontorium
3.	Anteroposterior pelvic outlet diameter (APOD)	Distance between inferior pubic symphysis and coccyx bone
4.	Conjugate pelvic inlet diameter (CPID)	Distance between superior pubic symphysis and sacral promontorium
5.	Left iliac breadth (LIB)	Distance between anterior superior iliac spine to superior posterior iliac spine (taken from the left side)
6.	Left ischium length (LIL)	Distance between the innermost point of ischial tuberosity and acetabular junction (taken from the left side)
7.	Left pubic length (LPL)	Distance between the superior point at pubic symphysis and acetabular junction (taken from the left side)
8.	Left width of greater sciatic notch (LGSN)	The line between iliac spine, the innermost part of greater sciatic notch, and ischial spine (taken from the left side)
9.	Left innominate height (LIH)	Distance between the most superior point of iliac crest and the lowest point of ischial tuberosity (taken from the left side)
10.	Pubic symphysis length (PSL)	Distance between the most superior and inferior points of pubic symphysis (taken from the left side)
11.	Right ischium length (RIL)	Distance between the innermost point of ischial tuberosity and acetabular junction (taken from the right side)
12.	Right pubic length (RPL)	Distance between the superior point at pubic symphysis and acetabular junction (taken from the right side)
13.	Right width of greater sciatic notch (RGSN)	The line between iliac spine, the innermost part of greater sciatic notch, and ischial spine (taken from the right side)
14.	Right iliac breadth (RIB)	Distance between anterior superior iliac spine to superior posterior iliac spine (taken from the right side)
15.	Right innominate height (RIH)	Distance between the most superior point of iliac crest and the lowest point of ischial tuberosity (taken from the right side)
16.	Sub pubic angle (SPA)	The angle formed by the point of iliac spine, the innermost part of great sciatic notch and ischial spine
17.	Transverse diameter of sacral segment1 (TDSS)	Distance between the two most lateral points of the first sacral segment
18.	Transverse pelvic inlet (TPI)	Distance between coccyx and inferior pubic symphysis
19.	Transverse pelvic outlet (TPO)	Distance between sacral promontorium and superior pubic symphysis
20.	Left IschPub index	Pubic length (× 100) divided with ishcial length

### Statistical analysis

Data analysis was performed using SPSS 23 statistics software. The mean of radiometric value from each observer was tested for inter-observer agreement. The inter-observer agreement was evaluated by Cohen’s kappa coefficient test, where *p* < 0.05. T-test analysis was used when the data was parametric; however, Mann–Whitney test was used when the data was non-parametric. The purpose was to determine a significant difference between sexes. The correlation strength analysis was conducted using eta test if there were significant differences. Moreover, the variable with the strongest correlation was tested to find the regression formula to determine an adult’s sex. Then, after the formula was determined, the variable was used to test the second observation group. Afterward, the validity and specificity were measured. Chi square test was conducted to determine correlation relationship.

## Results

### Study sample demography

There were 204 patients who were included in this study. Most of the samples were male with female-to-male ratio of 1:1.13. Mean age of the patients was 50.23 ± 14.36 years old and the mean age of male group is older than female group. Patient’s age was divided according to age group, with an age range of 10 years for each group. Group with the most patients was 41–50 years age group and followed by 51–60 years age group. Most men were included in 51–60 years age group, while most females were included in 41–50 years age group (Table 2).

**Table 2:** Age group of patients

**Age group (years)**	**Sex**		**Total**
	**Male**	**Female**	
11–20	2	3	5
21–30	7	10	17
31–40	10	11	21
41–50	27	32	59
51–60	31	16	47
61–70	25	21	46
71–80	3	1	4
81–90	3	2	5

### Radiologic components

**Fig. 1:**
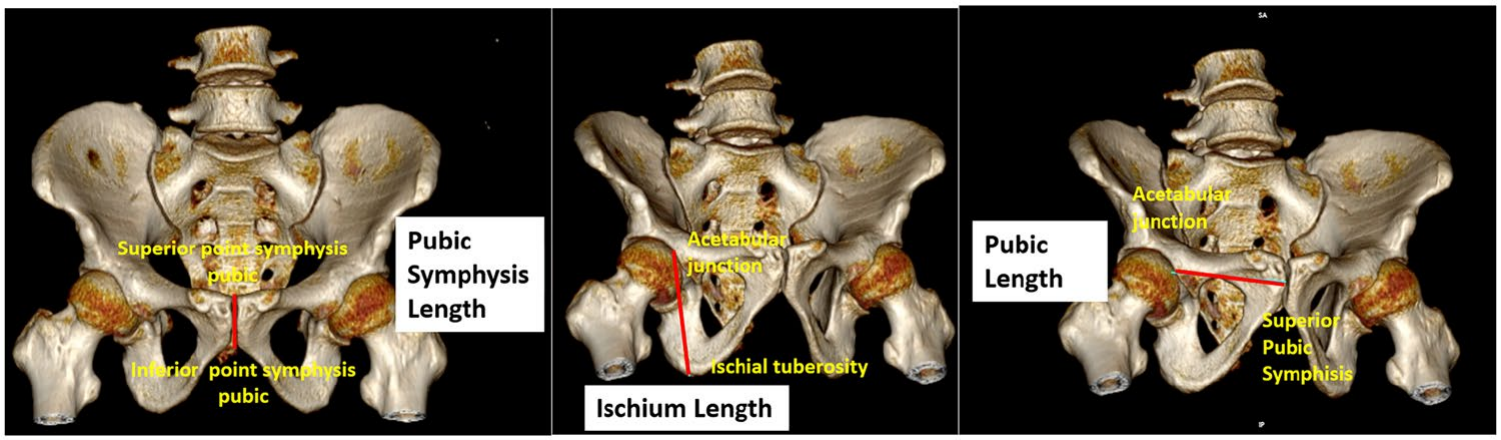
Illustration of measuring the pubic symphysis length, ischium length, pubic length on 3D model of pelvic CT. Pubic symphysis length (PSL) is measured by distance between the most superior and inferior points, ischium length (IL) is measured by distance between the innermost point of ischial tuberosity and acetabular junction, as well as pubic length (PL) is measured by distance between the superior point at pubic symphysis and acetabular junction

All radiologic components consisted of pelvic measurement results. The Cohen’s kappa test was carried out to determine the inter-observer agreement on the radiometric value’s measurement with the obtained *p* = 0.000 (*p* < 0.05) and the calculated Cohen’s Kappa coefficient (*κ*) was determined to be 0.856. These results indicated that the two observers have strong agreement in the evaluation of all radiometric values. Pelvic radiometric measurement values were taken for the entire sample. Data were presented in the form of mean and standard deviation and further divided by sex. Table 3 provided pelvic measurement results. Several variables were found to have a greater size for men than women, i.e., ABS, AHS, bilateral IB, bilateral IH, RIL, PSL. Furthermore, the data were divided based on each component of pelvic measurements obtained as in Fig. 1.

**Table 3:** The mean and standard deviation of radiometric components

**Radiologic components**	**Male**		**Female**		***P* value**
	**Mean**	**Standard deviation**	**Mean**	**Standard deviation**	
Anterior breadth of the sacrum (ABS)	95.89	7.90	91.98	13.44	0.004^a^
Anterior height of sacrum (AHS)	102.86	8.99	100.25	9.00	0.034^b^
Anteroposterior pelvic outlet diameter (APOD)	82.81	8.21	88.58	9.21	< 0.001^b^
Conjugate pelvic inlet diameter (CPID)	107.44	7.78	120.68	35.77	< 0.001^a^
Left iliac breadth (LIB)	116.97	9.79	113.85	11.59	< 0.001^a^
Left ischium length (LIL)	79.05	6.03	75.10	4.61	< 0.001^a^
Left pubic length (LPL)	66.04	6.25	66.79	4.13	0.021^a^
Left width of greater sciatic notch (LGSN)	43.89	13.63	47.84	4.97	< 0.001^a^
Left innominate height (LIH)	197.53	7.91	178.66	20.03	< 0.001^a^
Pubic symphysis length (PSL)	29.71	3.45	28.46	3.00	0.011^a^
Right ischium length (RIL)	79.29	5.82	73.73	10.84	< 0.001^a^
Right pubis length (RPL)	65.65	3.80	68.60	21.21	0.024^a^
R width of greater sciatic notch (RGSN)	42.84	4.19	47.16	7.00	< 0.001^a^
Right iliac breadth (RIB)	117.69	5.28	112.70	9.24	< 0.001^a^
Right innominate height (RIH)	195.96	18.81	178.96	11.54	< 0.001^a^
Sub pubic angle (SPA)	99.69	11.78	128.01	14.43	< 0.001^a^
Transverse diameter of sacral segment 1 (TDSS)	106.87	5.21	108.20	6.81	0.180^b*^
Transverse pelvic inlet (TPI)	114.03	6.64	122.48	6.52	< 0.001^b^
Transverse pelvic outlet (TPO)	94.00	7.64	108.90	9.57	< 0.001^a^
Left IschPub index (LIPI)	84.02	11.26	89.12	5.80	< 0.001^a^

^*^not significant

^a^Non-parametric difference test using Mann–Whitney test

^b^parametric difference test using t-test

In this study, 19 out of 20 variables had significant differences (*p* < 0.05) between male and female groups. One variable that did not have a significant difference between male and female groups was transverse variable diameter of sacral segment 1 variable (*p* = 0.180). Variables that have significant differences were analyzed for their correlation strength using eta test. The results of the analysis can be seen in Table 4.

**Table 4:** Correlation strength test between variables with eta test^*^

**Radiometric components**	**Eta value**	***p***
Right iliac breadth (RIB)	0.231	0.447
Pubic symphysis length (PSL)	0.705	0.733
Right pubis length (RPL)	0.751	0.529
Left pubic length (LPL)	0.816	0.363
R width of greater sciatic notch	0.834	0.289
Left ischium length (LIL)	0.838	0.290
Left width of greater sciatic notch	0.847	0.428
Right ischium length (RIL)	0.855	0.378
Left iliac breadth (LIB)	0.857	0.239
Transverse pelvic inlet (TPI)	0.859	0.434
Anterior breadth of the sacrum (ABS)	0.883	0.427
Left IschPub index	0.893	0.331
Anterior height of sacrum (AHS)	0.899	0.428
Right innominate height (RIH)	0.926	0.245
Anteroposterior pelvic outlet diameter (APOD)	0.955	0.154
Conjugate pelvic inlet diameter (CPID)	0.945	0.302
Left innominate height (LIH)	0.960	0.183
Transverse pelvic outlet (TPO)	0.972	0.120
Sub pubic angle (SPA)	0.982	0.204

^*^The eta correlation test is used to measure the association between interval and nominal variables. This can also measure of effect size that is commonly used in ANOVA models

In this case, the highest correlation between variables was RIH, APOD, CPID, LIH, TPO, and SPA. The innominate height variable had a high correlation strength for both sides (left and right); therefore, the authors decided to take the left side of innominate height (LIH) of pelvis to avoid result duplication and artificial inflation of the calculation results in making statistical models. Variables that used for making statistical models with logistic regression were APOD, CPID, LIH, TPO, and SPA.

### Making statistical models with logistic regression statistical analysis

Multivariate calculations were performed using binomial logistic regression on four variables with the highest correlation strength. Logistic regression calculations are presented in Table 5. From Table 5, it can be seen that the variables with a significant value for further use in logistic regression formula model from calibration group were CPID (*p* = 0.035), LIH (*p* = 0.001), and SPA (*p* = 0.015).

**Table 5:** Logistic regression analysis of APOD, CPID, LIH, SPA, and TPO in calibration group

**Variables**	***β***	**Wald**^a^	***p***	**OR**^b^
APOD	0.038	0.678	0.410	1.038
CPID	0.125	4.443	0.035	1.133
LIH	−0.180	16.243	0.001	0.835
SPA	0.078	5.901	0.015	1.081
TPO	−0.007	4.443	0.864	0.993
Constant	8.912	0.976	0.323	7423.517

^a^Wald test is used to analyze the association between the independent variables (predictors) and the criterion variable (dependent) variable

^b^OR is an abbreviation from outcome regression

The formula generated by logistic regression model from calibration group was obtained as follows:

$$\begin{aligned}\mathbf{Sex}\,=&\;\boldsymbol{(0.125}\:\times\:\mathbf{CPID}\boldsymbol{)}\;-\;\boldsymbol{(0.180}\:\times\:\mathbf{LIH}\boldsymbol{)}\:+\:\boldsymbol{(0.078}\:\times\:\mathbf{SPA}\boldsymbol{)}\\&+\:\boldsymbol{8.912}\end{aligned}$$

If the result > 0, the predicted sex is female; whereas, if the result < 0, the predicted sex is male.

## Logistic regression model validity

**Fig. 2:**
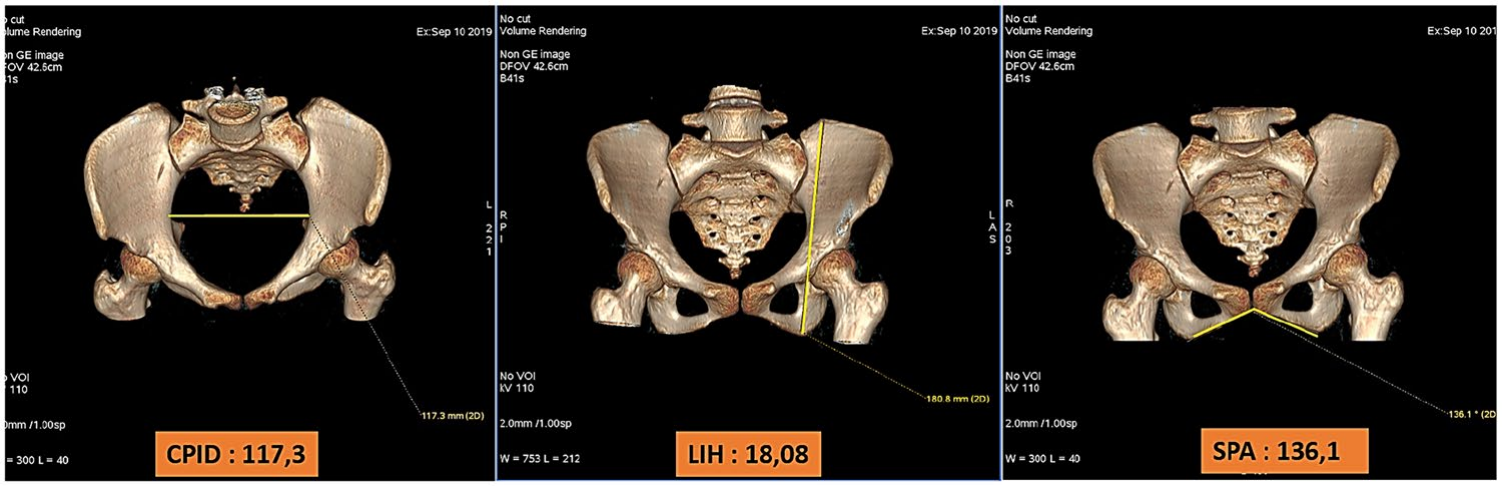
A case illustration of applying the regression formula using 3D pelvic CT anthropometric parameters in 43-year-old female patient. A total calculated value is 30.9, consistent with the sex of female pelvic

Logistic regression model was applied in the studied groups to determine the formula accuracy. Data of model validity were presented in Table 6. From logistic regression model calculation, a high validity value (91.05% accuracy) was found with 100% sensitivity to identify male and 81.1% specificity to identify female. A case illustration of using the regression formula in 43-year-old female is described in Fig. 2.

**Table 6:** Logistic regression formula validity in determining sex of the studied groups

**Sex**	**Correct sex**		***n***	***p***
	**Male**	**Female**		
*Sex prediction according to formula*				< 0.001
Male	40	5	45	
Female	0	23	23	
**Total**	40	28	68	
Sensitivity	100%			
Specificity		81.1%		
Total accuracy	91.05%			

91.05% accuracy was found with 100% sensitivity to identify male and 81.1% specificity to identify female

## Discussion

Geographically and genetically isolated human groups were the sources of matric sex determination. One of them was based on display population-specific skeletal characteristics. Many of which are evident in the relative expression and magnitude of sexually dimorphic features [15, 16]. In fact, each population has its special identification standard. Implementing visual or morphological techniques is the quick way to assess the samples. The weakness of this technique is in the sense of a very subjective assessment which requires an experienced observers or assessors and the level of accuracy is not guaranteed. Moreover, sexual dimorphism varies geographically. Therefore, forensic anthropologists are constantly trying to test the existing methods and developing standards that are more efficient and objective in which it can optimize the positive identification of the human skeleton. Numerous attempts at metric classification have been published, but often require complex or time-consuming measurements. However, while nonmetric methods are a quick means of assessment, they tend to be extremely subjective [15–19].

This study demonstrated the importance of an objective method utilizing radiological technology in human anthropometric determination in order to assist the analysis of sex determination. Medical image data provide the opportunity for high-end forensic analysis to be conducted outside the usual confines of traditional anthropological procedures. Imaging modalities, e.g., CT, are extensively used in the diagnosis and treatment of patients in a clinical setting. Murail and his colleagues have developed a database called Probabilistic Sex Diagnosis (DSP: Diagnose Sexuelle Probabiliste) in 2005, a sex determination method based on a worldwide hip bone metrical database. CT scans of bones were then analyzed to obtain three-dimensional (3D) virtual models then importing the models into a customized software program. CT scan imaging of the above dry bones was performed to obtain three-dimensional (3D) virtual models. 3D models were obtained using a commercial software (Amira) allowing semi-automatic segmentation [6, 20].

This study utilized medical image data from a 16-slice CT scanner. The speed of CT and its ability were applied to demonstrate bony features without the need for soft tissue removal, in the extent to make it an ideal modality to reduce time evaluation and could avoid physical manipulation. The virtual model of the pelvis can be analyzed out of contact with the actual bone. Therefore, the speed of the resulting virtual bone model and measurements make this method a practical alternative to traditional analyses [1–3, 10–12, 21, 22]. This study was undertaken to investigate whether three-dimensional (3D) volumetric virtual models can be used in the estimation of sex from the pelvis and if they can, whether “metricizing” nonmetric sex estimation traits in the pelvis and utilizing current medicine indices will increase the accuracy and reliability of the data over current methods [3, 4, 10, 12, 23, 24].

As a matter of fact, Asia consists of many countries and ethnics. This extent could result in many variations of body size. Unfortunately, the anthropometric studies that had been published more likely discuss the coverage of European, North American, and African population. Only few studies portrayed the characteristic data of Asians. In this study, the population of the eastern part of Indonesia is expected to be a population representing the pelvic shape of the Indonesian population.

The mean age of sample in this study was 50.23 ± 14.36 years. Moreover, in a study conducted by Kolesova et al., the pelvic size difference was associated with changes in age. Age-related changes observed in the study were carried out in linear parameters of pelvic cavity and confirmed the anterior tilt of sacral floor as well as more horizontal sacrum location relating to aging. This study also showed that there was no change in pelvic proportion to ischial height in female, while the distance of transverse pelvic diameter shortens with age [21]. As it is stated previously, age-related ankylotic processes decreased sacroiliac joint motility and facilitated these changes [22, 23, 25].

There were significant differences (*p* < 0.05) on radiologic components measured between males and females except for transverse diameter of the sacral segment (*p* = 0.180). These significant difference finding was similar to other studies in different populations which there were significant differences in pelvic measurements between the sexes [4, 25]. In a study by Patriquin et al., they demonstrated significant sexual dysmorphism in a population study on South Africa. This study reported differences in pelvic size between sexes as well as differences between races [25].

Furthermore, this study showed a significant difference in APOD measurement between male and female groups. This result was in accordance to a study conducted by Kolesova and Vetra that there was significant difference in APOD measurements of the two sexes [26]. The result obtained from our study provided a lower mean of APOD value than their study, but it was similar in the sense of APOD value for males due to the fact that it had lower mean than females. The measurement of CPID component in this study showed a higher value in female group.

In previous literature, male pelvic structure is significantly heavier and thicker than females. The male pelvis is also adjusted to fit in more massive and sturdy body architecture, e.g., the male acetabulum has been designed to fit a larger femur. Even though most of pelvic sexual dimorphisms are due to size differences, sex-related shape variations are also very striking and cannot be considered an allometric difference in body size between both sexes [27]. This variation in shape was indicated by a rounder frame of female pelvis. Sciatic indentation was wider in the sense of females. They have larger, shallower, lower, bigger pelvis and larger pelvic inlet and outlet (pubic bone is longer and curvature degree of pectineal line is greater). Therefore, women’s pelvic bones also differ in characteristics related to sacroiliac joint position on the iliac bones [28].

The SPA measurement showed a significant difference between male and female groups with high correlation strength. This result was in accordance to previous studies which concluded that SPA was the most reliable indicator of pelvic sex [29]. This was also in accordance to a study by Igbigbi and Msamati, who stated that the accuracy of SPA dimensions in determining sex was 94.7% for females and 95.5% for males [30]. Moreover, a similar result was also portrayed in a study by Mostafa, which showed a significant difference in SPA measurements between both sexes [24]. Women’s growths tend to increase during adolescence, especially in ischium and pubic areas, resulting in a larger pelvic outlet, longer pubic, and a blunter SPA. This growth difference was related to sexual dimorphism associated with birth process [31, 32].

The TPO measurement showed a significant difference in both sexes with high strength of correlation between male and female groups. These findings were in accordance to previous studies which concluded that a significant difference was observed in transverse diameter of pelvic midplane and outlet. This could be explained by hormonal effects of pregnancy which result in pubic symphysis softening and pubic bone movement as wide as 1 cm, as a consequence there was an increase on pelvic diameter [24, 26, 31, 32]. Females tend to exhibit an increase in growth during the adolescent growth phase in the region of the pubis and ischium, which results in a longer pubis, a larger pelvic outlet, and a more obtuse subpubic angle. These differences in growth are related to the sexual dimorphism between males and females associated with parturition [31].

The significant differences in the radiological components of the pelvis measured in this study have desirable value in determining sex; however, we believed that using all components of the pelvic measurement as a sex-determining formula for the human skeleton is an inefficient concept. Therefore, the correlation strength test was carried out with the ETA test where it was seen that the variables had higher power than the others. The APOD, CPID, LIH, SPA, and TPO were conducted through multivariate analysis using logistic regression in order to find significant variables and generate a formula that might determine a person’s sex with high accuracy [32]. The CPID, LIH, and SPA components showed significant values in accordance to previous discussion. These three components consistently showed differences in sexual dimorphism in both sexes, especially SPA served as one of the high scoring factors consistently. The APOD, CPID, LIH, SPA, and TPO were conducted through multivariate analysis using logistic regression in order to find significant variables and generate a formula that might determine a person’s sex with high accuracy [32]. The CPID, LIH, and SPA components showed significant values in accordance to previous discussion. These three components consistently showed differences in sexual dimorphism in both sexes, especially SPA served as one of the high scoring factors consistently. This formula was able to provide a high overall validity (91.05% accuracy) with 100% sensitivity for male identification and 81.1% specificity for female identification. Nevertheless, these results need to be compared with previous studies in which they were able to provide higher validity values that could reach 100% [3, 24]. This study showed that the estimated values of pelvic measurements using 3D-CT could give birth to a pelvic model with a formula that has a high accuracy value using CPID, LIH, and SPA values. These specific radiometric parameter obtained from this study has a positive impact for analyzing and describing the pelvic shape and size among male and female group population.

This study had some limitations in the extent of a complete and intact pelvis CT data; however, in some instance, the actual case remains that have been examined in the anthropological setting are incomplete or fragmented pelvic bone. Furthermore, the important aspect by taking advantage 3D software that could reconstruct the bone fragmentation in virtual space and the anthropometric measurement is highly possible to deliver in determining the human sex. Multicentric studies were needed to obtain a greater variety of data and produce more accurate data and formulas. In addition, an analytical study of previously published studies could compare differences in pelvic anthropometric values from different races and geographic areas.

## Conclusion

There were differences on radiometric variable characteristics in adult Indonesian’s pelvic anthropometric study at Dr. Soetomo General Academic Hospital, Surabaya. The APOD, CPID, LIH, TPO, and SPA had the highest correlation between both sexes and had the greatest power than other components of the pelvic measurement for sex determination. The estimated values of pelvic measurements by using 3D CT images could develop a pelvic model with a regression formula with high accuracy value using CPID, LIH, and SPA values. The regression formula for sex determination on adults using 3D CT produces validity of 91.05%, sensitivity of 100% for male identification, and specificity of 81.1% for female identification.

## Key Points

1. There were differences on radiometric variable characteristics in pelvic anthropometric study among adult Indonesian at Dr. Soetomo General Academic Hospital, Surabaya.
2. A pelvic model is developed by pelvic measurements by using 3D CT images and resulting in a regression formula with high accuracy on CPID, LIH, and SPA values.
3. There were significant differences (*p* < 0.05) on radiologic components measured between males and females except for transverse diameter of the sacral segment (*p* = 0.180)
4. The regression formula for sex determination on adults using 3D CT produces validity of 91.05%, sensitivity of 100% for male identification, and specificity of 81.1% for female identification.

## Data Availability

The data that support the findings of this study are available from the corresponding author.

## Abbreviations

ABS: Anterior breadth of the sacrum
AHS: Anterior height of sacrum
APOD: Anteroposterior pelvic outlet diameter
CPID: Conjugate pelvic inlet diameter
LIB: Left iliac breadth
LIL: Left ischium length
LPL: Left pubic length
LGSN: Left width of greater sciatic notch
LIH: Left innominate height
MIP: Maximum intensity projection
NMDS: Non-metric multidimensional scaling
PCoA: Principal coordinates analysis
PSL: Pubic symphysis length
RIL: Right ischium length
RPL: Right pubis length
PL: Pubic length
RGSN: Right width of greater sciatic notch
RIH: Right iliac breadth
RIH: Right innominate height
SPA: Sub pubic angle
TDSS: Transverse diameter of sacral segment 1
TPI: Transverse pelvic inlet
TPO: Transverse pelvic outlet
CT: Computed tomography
MRI: Magnetic resonance imaging
VR: Volume rendering
